# Die „historische Studie“ SOLIDARITY als Antwort der Forschung auf die Sars-CoV-2 Pandemie

**DOI:** 10.1007/s00048-020-00257-5

**Published:** 2020-05-12

**Authors:** Mariacarla Gadebusch Bondio, Maria Marloth

**Affiliations:** 1 Institute for Medical Humanities, UKB University Hospital Bonn, Sigmund-Freud-Str. 25, 53127 Bonn, Deutschland; 2 University of Cologne, Faculty of Medicine and University Hospital Cologne, Department of Psychiatry, Germany, University of Cologne, Köln, Deutschland

**Keywords:** COVID-19, Forschungsethik, Evidenzbasierte Medizin, SOLIDARITY, WHO, COVID-19, Research ethics, Evidence-based medicine, SOLIDARITY, WHO

## Abstract

Dieser Beitrag ist Teil des Forums COVID-19: Perspektiven in den Geistes- und Sozialwissenschaften. Das neuartige Coronavirus (Sars-CoV-2) stellt die Weltgemeinschaft vor eine große Herausforderung. Das Wissen über das Virus und seine Eigenschaften ist lückenhaft, aber der Bedarf, politische und medizinische Entscheidungen an wissenschaftlicher Erkenntnis auszurichten ist groß. Diese Lage führt zu einer Dynamisierung der Forschung. Ein prominentes Beispiel ist die WHO-Studie SOLIDARITY. Die epistemologischen Besonderheiten und die daraus resultierenden ethischen Implikationen werden in diesem Beitrag näher beleuchtet.

## Abstract (en)

This paper is part of Forum COVID-19: Perspectives in the Humanities and Social Sciences. The novel coronavirus (Sars-CoV-2) poses a huge challenge to the world community. Knowledge about the virus and its properties is limited, but there is a great need to base political and medical decisions on scientific knowledge. This situation is leading to a dynamization of research. A prominent example of such a development is SOLIDARITY. The epistemological dimensions of this trial, which is coordinated by the WHO, and the resulting ethical implications are discussed in this article.

Die Geschwindigkeit der globalen Ausbreitung des Coronavirus (Sars-CoV-2) setzt die Wissenschaft unter Zeitdruck. Anders als bei Infektionsausbrüchen wie HIV/AIDS, Ebola und SARS ist die Dystopie einer sich rasant über den ganzen Globus ausbreitenden Infektion mit letalen Folgen für Tausende von Menschen Realität geworden. Wirksame Maßnahmen müssen gut überlegt, schnell entschieden und umgesetzt werden. Dabei ist über das Virus bisher zu wenig bekannt (Paules et al. 2020). Verunsicherung ist mit lückenhaftem Wissen verknüpft (Ioannidis 2020). Auf politischer Ebene ist der moralische Imperativ, Leben zu schützen, mit der Ungewissheit gepaart, die die Grenzen der Medizin und der Versorgungslage bedingen. Um klinisch effektiv zu handeln, braucht die Medizin zuverlässige Daten und robuste Evidenz. Auch Gesellschaft und Politik benötigen wissenschaftliche Evidenz, um einschneidende Maßnahmen zu legitimieren. Der US-Virologe Anthony Fauci und seine Ko-Autoren^1^ verstehen Sars-CoV‑2 als

[A] stark reminder of the ongoing challenge of emerging and reemerging infectious pathogens and the need for constant surveillance, prompt diagnosis, and robust research to understand the basic biology of new organisms and our susceptibilities to them, as well as to develop effective countermeasures. (Fauci et al. 2020).

Dieser dringende Bedarf an evidenzbasierten Lösungsansätzen führt zu einer Dynamisierung der Forschung, deren epistemische und ethische Dimensionen im Folgenden skizziert werden.

## SOLIDARITY – „an historic trial“

Im Kontext einer weltweiten Gesundheitsherausforderung, die die WHO als „Public Health Emergency of International Concern (PHEIC)“^2^ und später als Pandemie klassifiziert hat, wird die Wissenschaft aufgefordert, ihre üblichen Kommunikationsmodi zu ändern und Studiendesigns an die Situation zu adaptieren.

Relevante Forschungsdaten sollen noch vor ihrer Veröffentlichung zirkulieren, eine Initiative, die das „International Comitee of Medical Journal Editors“ befürwortet (Moorthy et al. 2020). Viele Publikationen werden künftig verfügbar sein, bevor sie einen Peer-Review-Prozess durchlaufen. Eine kritische Evaluierung der Literatur muss durch die Leser vorgenommen werden, was eine gesteigerte Eigenverantwortung bedeutet. Schließlich verspricht der ungehinderte Datenfluss die laufende Forschung zu beschleunigen. Politische und gesellschaftliche Entscheidungsprozesse können so schrittweise justiert werden. Bereits validierte Evidenz wird in den Datenbanken gebündelt und zur Verfügung gestellt.^2^ Die Dringlichkeit, therapeutische Ansätze zu entwickeln und zu validieren, ist durch die Geschwindigkeit der Ausbreitung von SARS-COV‑2 deutlich verschärft. Von einer epistemisch und ethisch bemerkenswerten Entwicklung zeugt in diesem Kontext die von der WHO lancierte SOLIDARITY-Studie (WHO 2020), ein „Megatrial“, an dem 45 Länder beteiligt sind (Kupferschmidt & Cohen 2020). Medikamente zur Behandlung von SARS-COV‑2 sollen in dieser Studie weltweit getestet werden. Dabei handelt es sich nicht um speziell dafür entwickelte Medikamente, sondern um das sogenannte *repurposing*-Verfahren. Das heißt, es werden bereits existierende, teilweise für andere Krankheiten zugelassene oder kurz vor der Zulassung stehende Medikamente auf ihre Wirksamkeit gegen SARS-COV‑2 getestet (Hanging et al. 2018; Newman 2018).^3^

Am 27. März 2020 gab Tedros Adhanom Ghebreyesus, Generaldirektor der WHO, dieses Vorgehen bekannt: „This is a historic trial which will dramatically cut the time needed to generate robust evidence about what drugs work.“^4^

Das Studiendesign habe nach seiner Einschätzung dieses Potential, wobei die angewendete Methode keine neue ist. Ein Blick auf die Forschung zu HIV/AIDS, Ebola und SARS zeigt, dass der dringende Bedarf nach einer Therapie zu vergleichbaren Strategien geführt hat (Bai & Hsu 2019; Zhou et al. 2020). Eindrücklich ist, dass bereits Mitte der 1980er Jahre im Zusammenhang mit der HIV/AIDS Pandemie die klinische Forschung erfolgreich das sorgenannte „drug repurposing“ erprobte (Killen 2008). Bis heute ist *drug repurposing* eine gute Alternative, wirksame Therapien für neu auftretende Erkrankungen zu identifizieren, da für die Entwicklung eines neuen Medikaments im Durchschnitt 13–15 Jahre notwendig sind. Gelingt es, Medikamente zu finden, für die eine neue therapeutische Indikation validiert werden kann, erspart man sich lange, risikobehaftete und kostenintensive präklinische und frühe klinische Phasen (Pushpakom et al. 2019). Für *repurposing*-Verfahren können auch *sleeping candidates* in Erwägung gezogen werden. Es handelt sich dabei um Medikamente, die während des Entwicklungsprozesses und bei fortgeschrittenen Phasen der klinischen Prüfung aufgegeben wurden (Phase II und III), da sie sich als unzureichend wirksam für die ursprünglich gedachte medizinische Anwendung erwiesen haben bzw. mit Nebenwirkungen behaftet sind. Eine neue therapeutische Indikation könnte potentiell solche Kandidaten „wiederbeleben“ (Hernandez et al. 2017). Ein historisches Beispiel für einen *sleeping candidate* ist AZT (Zidovudine). Als Krebsmedikament entwickelt erwies es sich als ineffektiv. Allerdings bestätigte sich 1986 in einer Placebo-kontrollierten-Studie (RCT) die Vermutung, dass es wirksam gegen AIDS eingesetzt werden könnte. 1987 wurde AZT als erstes antiretrovirales Medikament zugelassen (Fauci 2003). Die Dringlichkeit einer pandemischen Situation, in der eine potentiell tödliche „Seuche“ die Wissenschaft vor die Aufgabe stellt, schnell eine Therapie zu entwickeln, bildet die Voraussetzung für die Prüfung von *sleeping candidates *oder von Medikamenten, die bereits für andere Erkrankungen etabliert sind. Doch anders als bei Ebola betrifft das Coronavirus die Weltgemeinschaft. Um – trotz Überbelastung – zeitgleich so viele Zentren wie möglich an der Studie zu beteiligen, wurde das Studiendesign von SOLIDARITY schlank gehalten. Von der Patientenaufnahme, Aufklärung und Einwilligung, bis hin zur Dokumentation sind alle Schritte pragmatisch und programmunterstützt.

Aus epistemischer Perspektive ist hervorzuheben, dass die WHO sich aufgrund des Zeitdrucks und der rasant steigenden Opferzahlen nicht nur für ein schlankes, sondern auch dynamisches Studiendesign entschieden hat. Anpassungen sind während der Studie jederzeit möglich. Zwar handelt es sich um eine RCT, aber ohne Verblindung und ohne Placebogruppe. Im Laufe der Studie wird ein Komitee die gesammelten Daten überwachen. Sollte sich zeigen, dass es keinen Effekt hat, kann man gegebenenfalls eines der vier Mittel aus der Testung nehmen. Auch besteht die Möglichkeit im Verlauf der Studie weitere Medikamente aufzunehmen.^5^ Das flexible Studiendesign ist mit dem Ziel verbunden, Patienten eine lebensrettende Therapie möglichst rasch anzubieten. Aus ethischer Sicht nimmt man mit dieser Zielsetzung eine schwächere Evidenz in Kauf. Die Autoren eines Berichts über SOLIDARITY betonen, dass dies den Koordinatoren der Studie bei der WHO bewusst sei, „WHO says it had to balance scientific rigor against speed“ (Kupferschmidt & Cohen 2020). Neben der Inkaufnahme des erhöhten Risikos einer systematischen Verzerrung der Ergebnisse infolge fehlender Verblindung werden aufgrund des Zeitdrucks nicht wie sonst üblich alle Daten erfasst. So müssen beispielsweise Parameter, wie die Viruslast, nicht verfolgt werden. Diese Reduktion ermöglicht die Durchführung der Studie in unterschiedlichen Kontexten und ist forschungsethisch signifikant. Die Heterogenität der Studienpopulation entkräftet einen Einwand, der oft gegen RCTs ins Feld geführt wird. Zugunsten der Vergleichbarkeit bilden in der Regel die selektierten Studienteilnehmer eine sehr homogene Gruppe, deren Eigenschaften nicht denen der tatsächlichen Patienten entsprechen, die am Ende die Therapie erhalten. Studienteilnehmer weisen meistens weniger oder nicht so gravierende Komorbiditäten auf (Campbell-Scherer 2010). Gerade die Fokussierung auf männliche Studienteilnehmer hat zu Debatten in der Medizin und in der Medizinethik geführt (Gadebusch Bondio 2014). Das Studiendesign von SOLIDARITY unterliegt diesen Einschränkungen nicht. Die Kenntnis von den potentiellen Nebenwirkungen der Behandlungen begünstigt eine liberalere Einschlusspraxis. Schließlich stellen Strategien, Anpassungen und Kompromisslösungen, die es ermöglichen, klinische Studien im Umfeld eines Pandemieausbruchs vorzunehmen, ein forschungsethisch wenig bearbeitetes Feld dar. Umso wichtiger scheint es uns, an dieser Stelle an die wissenschaftlichen und ethischen Kriterien zu erinnern, die H. Clifford Lane und Koautoren in Anlehnung an vorangegangene Überlegungen von Emanuel et al. für die korrekte Studiendurchführung von experimentellen Therapien für HIV und Ebola aufgestellt haben (Lane et al. 2016; Emanuel et al. 2004). Diese sind:

1. Beachtung einer sorgfältigen Patientenaufklärung als Voraussetzung für das Einverständnis zur Studienteilnahme;
2. klare Definition der primären Endpunkte im Studiendesign;
3. gute Kommunikation und Kooperation der teilnehmenden Länder;
4. Etablierung einer Instanz, die die wissenschaftliche Arbeit überwacht;
5. Transparenz und zeitnahe Weitergabe von Daten an behandelnde Ärzte und betroffene Zielgruppen.

Auch für die gegenwärtige Situation im Kontext des Pandemieausbruchs bieten diese Kriterien einen wertvollen ethischen Orientierungsrahmen.

## Der Not eine Tugend?

SOLIDARITY profitiert von der Koordination und Unterstützung durch die WHO. Die breite Inklusion von Patienten und die flexible Studienarchitektur sind vielversprechende Eigenschaften. Wenn es gelingt, die gewonnenen Daten und die daraus extrapolierten Informationen den klinisch tätigen Ärzten und den Entscheidungsträgern zugänglich zu machen und alle Publikationen – wie geplant – im Open-Access-Format zu veröffentlichen, wird die internationale Gemeinschaft davon profitieren. Möglicherweise werden sich dann auch Entscheidungen sicherer treffen lassen. Tatsächlich könnte der Pandemieausbruch jene epistemischen Tugenden ankurbeln, die auch in „normalen“ Forschungskontexten wünschenswert sind: Anstatt interessengeleiteter Konkurrenz und Verschwendung von Zeit und Ressourcen würden Kooperation, Solidarität, Transparenz und Offenheit die Forschung kennzeichnen. Allen vom Virus betroffenen Ländern wird die Partizipation an SOLIDARITY ermöglicht. Die sonst in klassischen Studien vernachlässigten Gruppen einer Population werden inkludiert. SOLIDARITY ist dank ihres globalen Charakters eine „historische Studie“, doch methodologisch profitiert sie von den vorangegangenen pragmatischen Trials zur Entwicklung von dringend benötigten Therapien bei Epi- und Pandemien. Dies sind historische Beweise dafür, dass so konzipierte Forschung effektiver, gerechter und schneller sein kann.

## Footnotes

1 Zur besseren Lesbarkeit wird ausschließlich das generische Maskulinum verwendet. Es bezieht sich auf Personen aller Geschlechter.

2 Siehe in der Cochrane Library: https://www.cochranelibrary.com/Sars-CoV-2); und das Oxford SARS-COV‑2 Evidence Service des Oxford Centers of Evidence-Based Medicine: https://www.cebm.net/Sars-CoV-2/. Viele medizinische Fachzeitschriften verfügen über täglich aktualisierte Sonderbereiche zu SARS-COV‑2 (zum Beispiel NEJM, Lancet u. v. a.).

3 Im SOLIDARITY Trial werden untersucht: 1. das Virostatikum Remdesivir, das zur Bekämpfung des Ebola-Virus entwickelt wurde und intravenös verabreicht wird; 2. das Malaria-Medikament Chloroquin und Hydroxychloroquin, 3. die HIV-Kombination Lopinavir/Ritonavir – Kaletra genannt – und 4. diese Kombination ergänzt um Beta-Interferon

4 https://www.who.int/dg/speeches/detail/who-director-general-s-opening-remarks-at-the-media-briefing-on-Sars-CoV-2---27-march-2020

5 https://www.aerzteblatt.de/nachrichten/111267/WHO-testet-in-globaler-Studie-4-Therapeutika-auf-Wirkung-bei-SARS-COV-2
